# The use of anticoagulants for venous thrombo-embolism

**Published:** 2010-04

## Introduction

Pulmonary embolism is a life-threatening condition with mortality as high as 17.5% within three months of onset.^1^ Pulmonary embolism is also the most common preventable cause of hospital death. Despite these unacceptably high risks, the effective use of anticoagulants to prevent venous thrombo-embolisms (VTEs) remains inconsistent.

Communication and education around the risk of VTE as a result of major orthopaedic surgery, particularly hip- and kneereplacement surgery, remains a concern. Talib Abdool-Carrim, professor of vascular surgery at Milpark Hospital, says to date this communication has been inadequate. ‘I believe medical professionals need to be constantly reminded, as only about 50% of hospital doctors are giving out guidelines for prophylactics, and we need to practice in line with the ACCP and South African guidelines. However, the use of anticoagulants for these patients has increased from a specialist perspective’, Prof Abdool-Carrim says.

Orthopaedic surgeon Dick van der Jagt believes medical professionals in South Africa are constantly being informed about prophylaxis for these patients, but that this communication needs to be repeated on an ongoing basis to prevent complacency setting in. ‘Every patient needs to be assessed to determine their individual risk profile, and given appropriate prophylaxis against VTEs’, he says.

Prof Mervyn Mer, critical care specialist and one of the authors of the South African Guidelines for Prophylactic Anticoagulation agrees that communication around this issue needs to be optimised. ‘There’s no doubt that extended prophylaxis should be given to high-risk hip- and knee-replacement patients, and that it should be used in conjunction with mechanical devices such as pneumatic compressive devices which are applied to the lower limbs. These are a useful addition to prophylaxis agents as they assist in moving blood around and prevent stasis. These devices are not ideally optimal when used alone but do further decrease the chance of VTEs’, Prof Mer says.

According to Prof Abdool-Carrim, anticoagulants reduce the risk of VTEs by up to 80%. ‘There is enormous data to support the efficacy of anticoagulants in reducing the risk of VTE’, Prof Mer says. ‘It is not 100% guaranteed, but the risk is reduced so substantively that the omission of anticoagulants would compromise the medical practitioner and be difficult to defend. The use of anticoagulants to reduce the risk of VTE has become the number 1 focal point in US hospitals, with specialists being called upon to justify their actions should these agents be omitted.’

The new ACCP guidelines recommend the use of anticoagulants for a minimum of 10 days for knee-replacement surgery and up to 35 days for hip-replacement surgery. The new South African guidelines are fully aligned with the ACCP recommendations and have even extended the time period for knee-replacement surgery to two weeks. ‘In general, prophylaxis should be used whenever a patient is immobile, and should be continued until the patient is mobile again’, Prof Mer says.

According to Prof Abdool-Carrim, adequate prophylaxis is not always prescribed for hip and knee replacements or other major surgeries. Dr van der Jagt concurs, saying many patients only receive anticoagulants while in hospital. ‘However, this is often because medical aids will not cover the treatment outside of hospital care, and a high percentage of patients cannot afford to fund it themselves. The guidelines are clear, but unfortunately medical aids are more interested in their bottom lines’, he says.

The South African Guidelines for Prophylactic Anticoagulation allude to only currently registered agents, but are updated as the industry evolves. These guidelines have been endorsed by a number of societies including those of orthopaedic surgeons, vascular surgeons, anaesthesiologists, critical care specialists and pulmonary specialists. For the first time in South Africa, the guidelines include the input of two highly regarded international specialists in the field who have contributed independently to the guidelines to avoid any local bias.

Without anticoagulation treatment, patients undergoing major orthopaedic surgery have up to a 60% chance of developing a VTE.^1^ Recent advances in the field, however, indicate a brighter future as treatment has evolved from warfarin to intravenous heparin to the new oral anticoagulants which are due to be released onto the market soon.

According to Dr van der Jagt, the new oral anticoagulants offer a number of benefits over existing treatments: they are simple to administer, easy to manage and don’t need to be constantly monitored. In line with the trend to safely administer effective prophylaxis against VTEs, the oral agents also eliminate the need for injections and cut down on hospital costs as well as hospital-acquired infections by enabling patients to continue with the drugs at home.

Commenting on the future direction of these treatments, Prof Mer says the next step lies in the development of particular antidotes and the ability to measure the effects of these agents. ‘We need the ability to measure and monitor each patient’s requirements rather than using a fixed dose for most people’, he says. ‘The future for the prevention of VTEs is definitely looking positive’, Prof Abdool- Carrim added.

A further benefit of the new oral anticoagulant agents is that they offer minimal drug–drug and drug–food interactions, making the use of extended anticoagulation therapy more practical. ‘Some of the existing treatments do present a problem with drug–drug and drug–food interactions’, Dr van der Jagt says. ‘Choose a preferred oral anticoagulant which will effectively reduce the risk of clotting without severely increasing the risk of bleeding.’
